# Feasibility and Effectiveness of mHealth for Mobilizing Households for Indoor Residual Spraying to Prevent Malaria: A Case Study in Mali

**DOI:** 10.9745/GHSP-D-15-00381

**Published:** 2016-06-20

**Authors:** Keith Mangam, Elana Fiekowsky, Moussa Bagayoko, Laura Norris, Allison Belemvire, Rebecca Longhany, Christen Fornadel, Kristen George

**Affiliations:** a Abt Associates, U.S. President’s Malaria Initiative (PMI), Africa Indoor Residual Spraying (AIRS) Project, Bethesda, MD, USA; b Abt Associates, PMI AIRS Project, Bamako, Mali; c U.S. Agency for International Development, PMI, Washington, DC, USA

## Abstract

Components of mHealth are increasingly being added to development interventions worldwide. A particular case of interest is in Mali where the U.S. President’s Malaria Initiative (PMI) Africa Indoor Residual Spraying (AIRS) Project piloted a mobile mass-messaging service in Koulikoro District in August 2014 to determine whether voice and/or text messages received on cell phones could effectively replace door-to-door mobilization for an indoor residual spraying (IRS) campaign. To measure the pilot’s effectiveness, we evaluated structure preparedness (all household and food items removed) in 3 pilot intervention villages compared with 3 villages prepared for spray through door-to-door mobilization that was modified by incorporating town hall meetings and radio spots. Structure preparedness was significantly lower in households mobilized through the mobile-messaging approach compared with the door-to-door approach (49% vs. 75%, respectively; *P* = .03). Spray coverage of targeted households also was significantly lower among the mobile-messaging villages than the door-to-door mobilization villages (86% vs. 96%, respectively; *P* = .02). The mobile-messaging approach, at US$8.62 per structure prepared, was both more costly and less effective than the door-to-door approach at US$1.08 per structure prepared. While literacy and familiarity with technology were major obstacles, it also became clear that by removing the face-to-face interactions between mobilizers and household residents, individuals were not as trusting or understanding of the mobilization messages. These residents felt it was easier to ignore a text or voice message than to ignore a mobilizer who could provide reassurances and preparation support. In addition, men often received the mobile messages, as they typically owned the mobile phones, while women—who were more likely to be at home at the time of spray—usually interacted with the door-to-door mobilizers. Future attempts at using mHealth approaches for similar IRS mobilization efforts in Mali should be done in a way that combines mHealth tools with more common human-based interventions, rather than as a stand-alone approach, and should be designed with a gender lens in mind. The choice of software used for mass messaging should also be considered to find a local option that is both less expensive and perhaps more attuned to the local context than a U.S.-based software solution.

## Abstract (precis)

Sending voice and/or text messages to mobilize households for spraying was more costly per structure and less effective at preparing structures than traditional door-to-door mobilization approaches supplemented with radio and town hall announcements. Challenges included:

- Lack of familiarity with mobile phones and with public health mobile messaging
- Lack of face-to-face communication with mobilizers, making it easier to ignore mobilization messages and preventing trust-building
- Low literacy levels
- Gender differentials in access to mobile phones

## INTRODUCTION

The main strategy for lowering malaria prevalence is through vector control, which targets the mosquitoes that transmit malaria. The World Health Organization states the 2 most effective vector-control measures are long-lasting insecticide-treated nets (LLINs) and indoor residual spraying (IRS).^1^ This article focuses on IRS.

IRS is the application of insecticide to the inside of dwellings, on walls and other surfaces that serve as a resting place for mosquitoes that transmit malaria. The insecticide then kills mosquitoes as they come in contact with treated surfaces. In order for IRS to be most effective, the following conditions must apply:

1. Majority of vectors (i.e., mosquitoes that transmit malaria) must feed and rest indoors.
2. Vectors are susceptible to the insecticide in use.
3. Houses have surfaces that are eligible to be sprayed (i.e., porous, permanent surfaces).
4. A high proportion of the houses in target areas are sprayed (more than 80%).

Indoor residual spraying involves applying insecticide to the inside of dwellings to kill the mosquitoes that transmit malaria.

IRS campaigns include a period of community mobilization intended to educate residents about the causes and risks of malaria, ways to protect against the disease, and the benefits of IRS to prepare beneficiaries for household spraying and ensure the IRS campaign achieves at least 80% coverage—the point at which the full potential of IRS is realized.^1^ At 80% coverage, the 20% of households that have *not* received protective measures can benefit from the reduction in transmission achieved by the other 80% of households in the community. The initial periods of insecticide spraying are usually followed by a “mop-up” period to revisit areas where the overall number of structures sprayed was below the 80% target.

Traditionally, door-to-door mobilization methods are used to prepare structures for IRS and to ensure general understanding and acceptance of IRS campaigns. With the unprecedented growth in mobile phone users around the world, however, mHealth offers great potential for reaching a large proportion of populations with behavioral messages that address an array of public health challenges, including IRS mobilization.^2^ The number of mobile-cellular subscriptions has increased from just over 2 billion in 2005 to 6.9 billion globally in 2014, with low-income developing countries accounting for 78% of these subscriptions.^3^

Door-to-door mobilization methods are traditionally used to prepare households for indoor residual spraying, but mHealth may provide new approaches for reaching people.

A systematic review of mHealth projects in Africa illustrates that these initiatives have generated a number of positive results including: (a) support to patients to make health care appointments, (b) reduced communication delays, (c) improved data collection, (d) efficient communication with patients, resulting in reduced need for transportation and increased uptake of diagnostic services, and (e) improved health care provider compliance with protocols. Researchers have attributed these successes to accessibility, acceptance, and affordability of the technology used, as well as the ability to obtain stakeholder buy-in and to effectively integrate the use of tools into the local context.^4^ For example, evidence from studies in India and Peru indicate that both patients and health care workers considered text messages to be highly useful for appointment reminders and for communicating with health care providers and receiving health information.^5^

There is limited research documenting whether cost savings are generated by mHealth initiatives. However, it is acknowledged that these efforts have the potential to reduce costs related to the management of patient records, production of vital statistics, and referral and billing processes, by shifting from time-intensive paper-based processes to more efficient processes that make use of shared technological platforms. Similarly, since mHealth initiatives are able to reduce the need for physical displacement of patients and/or providers, they can result in a subsequent decrease in the time needed to provide those services. The strategy of cost savings needs to be evaluated in multiple scenarios to determine in which settings an mHealth approach could indeed decrease program costs.

The purpose of this case study was to evaluate the use of mHealth tools for IRS mobilization in Mali. The U.S. President’s Malaria Initiative (PMI) Africa Indoor Residual Spraying (AIRS) Project piloted a mobile mass-messaging service in 3 villages of Mali to determine whether voice and/or text messages received on cell phones could effectively replace door-to-door mobilization for an IRS campaign. To measure the pilot’s effectiveness, the team evaluated structure preparedness in the pilot villages and compared them with villages mobilized through a traditional door-to-door mobilization that also incorporated town hall meetings and radio spots. The case study’s goal was not only to evaluate the effectiveness of these mobile messages but also to determine if there was a reduction in mobilization costs.

## TRADITIONAL DOOR-TO-DOOR MOBILIZATION APPROACH USED IN MALI

The PMI AIRS Project in Mali traditionally uses a door-to-door IRS mobilization method in which, 30 days before an IRS campaign is set to begin, mobilizers inform households about the benefits of IRS, the date spray operators will be coming to their village, and what they need to do to prepare their homes for IRS.

For a household to be considered prepared, all furniture and belongings must be removed, as well as anything on the walls, from the rooms in which residents sleep. Similarly, any food products in the rooms must be taken outside and placed in a safe place until IRS has been completed. Any furniture or belongings that are too heavy to move must be covered with a plastic sheet that will protect them from any contamination by insecticide.

Households must prepare for IRS in advance by moving all furniture, belongings, and food from rooms in which residents sleep.

The PMI AIRS Project engages and trains mobilizers who work with the health facilities to deliver these IRS messages. In addition to the initial conversations with households in advance of the IRS campaign, these same mobilizers accompany the IRS teams during implementation. The mobilizers are able to assist the IRS team if a household has not properly removed belongings and furniture from within the house, and they can also help to encourage reluctant households to accept IRS.

Once a house has been sprayed, the mobilizers remind the beneficiaries of the guidelines they need to follow to prolong the effectiveness of the IRS and to ensure their safety. For example, all windows and doors must remain shut for 2 hours after IRS is completed, and then the rooms must be aired out for 1 more hour before anyone can enter. Then, once the necessary time has passed, a resident must enter the room and sweep up all the dead insects before being bringing all furniture and belongings back into the room.

In 2013, more than 1,000 mobilizers in Mali were used for these purposes in 3 districts implementing IRS. Because it is difficult to supervise such a large cadre of seasonal workers, performance quality varies widely. Furthermore, this method of door-to-door mobilization is expensive, both financially and in terms of human resources.

## METHODS

In an attempt to provide cost savings while maintaining structure preparedness, the PMI AIRS Project piloted a mobile-messaging mobilization approach in Mali’s Koulikoro District where the project has been implementing IRS since 2012. The purpose of this pilot was to effectively prepare the maximum number of structures for IRS, while reducing overall costs compared with traditional mobilization methods. To evaluate the feasibility of eliminating door-to-door mobilization, this project used only cell phone messaging for IRS mobilization in the pilot district. The hypothesis was that by reducing the number of people required to implement mobilization, and simultaneously removing the need for extensive field supervision of mobilizers, the project could benefit from a large reduction in mobilization costs while not diminishing the overall preparedness of structures. To measure the pilot’s effectiveness, the case study evaluated structure preparedness in the pilot intervention villages compared with preparedness in comparison villages mobilized through standard door-to-door mobilization methods. This activity was reviewed by the Institutional Review Board at Abt Associates and determined to be exempt from review.

### Intervention and Comparison Areas

**FIGURE 1:**
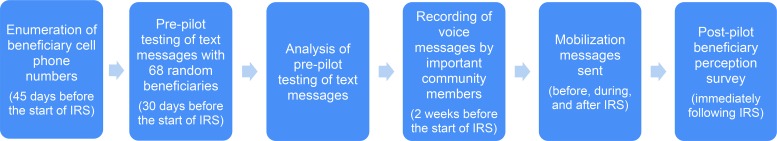
Flow of Activities for Mobile-Messaging Mobilization Approach, Koulikoro District, Mali, May–September 2014 Abbreviation: IRS, indoor residual spraying.

The mobile-messaging approach was piloted between May and September 2014 in 3 villages in the district of Koulikoro: Tienfala Village, Tienfala Gare, and Fougadougou. These villages were chosen because of their proximity to Bamako, with the assumption that a higher percentage of residents in these areas owned phones than residents living in areas farther from Bamako. Similarly, it was assumed that more of the residents were literate in the selected areas compared with those living in Bla or Baroueli, the other 2 IRS districts. Beneficiaries in all 3 intervention villages received text messages, and beneficiaries in Fougadougou and Tienfala Gare received voice messages in addition to text messages. Figure 1 demonstrates the flow of activities for this mobilization approach.

**FIGURE 2:**
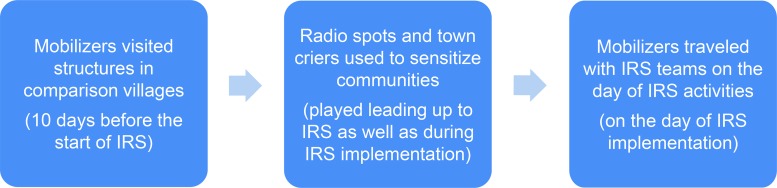
Flow of Activities for Door-to-Door Mobilization Approach, Koulikoro District, Mali, May–September 2014 Abbreviation: IRS, indoor residual spraying.

Three comparison villages were selected to receive the door-to-door mobilization in the district of Koulikoro: Fassa, Wolongotomo Socoura, and Wolongotomo Socoro. The door-to-door mobilization approach included: (1) door-to-door visits from mobilizers 10 days before the campaign, (2) radio spots on local radio stations explaining campaign information, (3) town hall meetings at the village level, and (4) a follow-up mobilizer to assist with IRS teams during implementation to encourage refusal cases to accept IRS and to help residents prepare their structures (Figure 2). Because this pilot added radio spots to prime beneficiaries about the benefits of IRS before mobilization teams arrived, the teams conducted door-to-door visits only 10 days before IRS began as opposed to the traditional 45 days before IRS.

### Enumeration of Cell Phone Numbers

The AIRS Mali monitoring and evaluation (M&E) team collected mobile phone numbers from 673 residents in 576 structures in the 3 pilot villages. The 576 structures do not represent the entirety of households in these communities. Some households were vacant during enumeration, and others did not have any phone linked to the household. Therefore, some households in the pilot villages were not included in the list of numbers that would later be used for sending text and voice messages.

After collecting phone numbers from the households in the 3 pilot villages, a data entry clerk uploaded the numbers into the TextIt platform, a commonly used technology platform for mass mobile messaging. The TextIt platform allows a single user to send short text messages to a large number of mobile phones via a computer. AIRS Mali used a team of data entry clerks to enter the collected mobile phone numbers into a database that allowed the M&E manager to run summary statistics on information about the individuals whose numbers had been collected, including the number of residents who were capable of sending text messages and who were literate in either French or Bambara (the local language). This helped the team determine which language to use when sending messages to specific groups of beneficiaries.

### Types of Mobile Messages

Messages were sent before, during, and after the IRS campaign. The AIRS Mali team sent 4 types of text and voice messages to beneficiaries:

- “Mobilization” messages were sent 10 days before the campaign to remind beneficiaries of the benefits of IRS and to let residents know the timing of the spray campaign in their village.
- “Alert” messages were sent 3 days before the start of the campaign to warn beneficiaries of the dangers of malaria and how they can protect themselves from malaria, such as accepting IRS.
- “Instruction” messages were sent during the spray campaign in advance of the arrival of IRS teams to let residents know what to do to prepare themselves and their structures for spraying, as well as what to do immediately following IRS.
- “Advice” messages were sent a few days after spraying to inform beneficiaries about what steps should be taken in order to prolong the positive effects of IRS, i.e., avoiding painting interior walls or hanging posters on treated surfaces.

Mobile messages were sent to households before, during, and after the campaign to educate and instruct households about IRS.

### Pre-Pilot Testing of Text Messages

Once the mobile phone numbers were registered in TextIt, the AIRS Mali team pretested the technology and the type of messages to see if they would be effective as a means for mobilization. The M&E team randomly selected 68 phone numbers (about 10% of the total number of phone numbers collected) to receive test messages. Messages were sent in Bambara and French, according to the self-reported preferences during enumeration of beneficiary cell phone numbers.

Two days after the messages were sent, the M&E team returned to the field to evaluate the perception and comprehension of these messages by 20 of the 68 beneficiaries who had received the test messages. The team chose only 20 beneficiaries due to time and resource constraints as well as to avoid delaying the start of the IRS campaign. Ultimately, only 18 of the 20 chosen beneficiaries were available for these short interviews.

Only 3 of the 20 individuals selected for interviews (15%) received and understood the text messages (Table 1). Conversely, 15 individuals (75%) were not able to use the information in the messages either because they were illiterate (n = 3), they did not read the message after receiving it (n = 11), or they deleted the message upon receipt without reading it (n = 1). Furthermore, only individuals with Android-based cell phones were able to see the Bambara characters on their screens. Simpler phones that are much more common in Mali cannot display the special characters contained in the Bambara alphabet. In this case, the Bambara letters are transliterated into the more common Latin alphabet, which is a common practice among simple phone users in Mali.

**TABLE 1:** Text Message Pretest Results for the Indoor Residual Spraying Campaign, Koulikoro District, Mali, May–September 2014

Village	Message Received and Understood	Message Received and Not Read	Beneficiary Unavailable at Time of Survey	Beneficiary Illiterate	Message Received and Deleted Before Reading	Total, No. (%)
Tienfala Gare	2	9	0	0	0	**11 (55)**
Tienfala Village	1	1	2	0	0	**4 (20)**
Fougadougou	0	1	0	3	1	**5 (25)**
**Total, No. (%)**	**3 (15)**	**11 (55)**	**2 (10)**	**3 (15)**	**1 (5)**	**20 (100)**

### Addition of Voice Messages

Based on findings from the pre-pilot text message testing, the team modified the methodology by adding voice messaging in the pilot to increase the possibility that beneficiaries would understand the mobile messages and be prepared for the spray campaign. Two of the pilot villages, Tienfala Gare and Fougadougou, received text messages plus voice messages, while the third pilot village, Tienfala Village, received only text messages, allowing the team to evaluate whether there were different performance outcomes based on the types of messages sent.

Voice messages were added to the mHealth approach to account for low literacy levels in the intervention areas.

The teams drafted scripts for the voice messages and then, in collaboration with the Health Center Technical Director (DTC) from Tienfala Gare, approached community representatives to record the messages (see Box for transcripts of sample voice messages). The team selected people who are well respected within the Tienfala Gare community, whose voices were recognizable, and who were well spoken: the village chief, an actress from Tienfala Gare, and the president of the community health associations (Association de Santé Communautaire, or ASACO). All voice messages were less than 1 minute long and recorded in French and Bambara in order to comply with beneficiary preferences. Due to time constraints, the team was unable to pretest the voice messages as they had done with the text messages.

**BOX. Sample Voice Message Transcripts for the Pilot Indoor Residual Spraying Campaign, Koulikoro District, Mali, August 2014**

**Table:** 

French	Bambara (Local Language)	English
**Sample Pre-Spray Messages**		
Les habitants de Tienfala ceci est un message pour vous informer que la campagne PID 2014 va commencer le 11 Aout 2014. Vous serez informé sur le calendrier de la campagne sur votre téléphone, les mesures à prendre avant, pendant et après la campagne. Tous unis pour une meilleure campagne de PID.	Anw Balima Tienfala Kaw so fiye baaraw bi da mine uti Kalo kile 11 Tienfala mara la. Kuna foni be di aw ma a ka telephoni ka campagni boloda bara hougoumou kono na waleya mounou kan ka ta sani campagne ka se ani campagne konona la ani ni campagne temena. Aw ni tie aw ka timinandiya la.	Residents of Tienfala, this message is to inform you that the Spray campaign will start on August 11^th^, 2014. You will be informed about the timing of the campaign on your phone, the measures to be taken before, during and after the campaign. All together for a better spray campaign.
Préparer votre structure à temps, tous les objets et meubles doivent être sortis de la structure ou rassemblés au centre et couverts afin de permettre à l’opérateur d’accéder facilement aux murs de la pièce.	Sokonominenw bee be labo kenema: tobiliminenw, finiw, dumininfenw, minen minuw dulo ne do kogow la.Minen girinmanw minnu talika gelen, olu be fara nogokan so cemance la, ku datugu kane. Obatofiyeli kela be seka sokono na soro nogoyala.	Prepare your structure at time, all objects and furniture must be removed from the structure and gathered in the center and covered to allow the spray operator easy access in the structure to spray.
La PID est gratuite, tous les frais sont pris en charge par l’USAID (fonds du peuple américain).	So fiye ye fu ye a moussaka be taledo lamericainw ka deme diekouloufe no bi wele ko USAID.	The Indoor Residual Spraying is free, all the costs are supported by USAID (funds of the American people).
**Sample Mid-Spray Message**		
Mettre tous les animaux domestiques en cage/dans un enclos hors de la maison, tenez éloigne les enfants pendant la pulvérisation.	Du denw bee ani du banga misenniw, ni chiyew bee be to kene ma fiyeli waati.Fiyelikela mana tila aw be so da tugou kane.	Put all animals out of the house and keep children away while spraying.
**Sample Post-Spray Messages**		
Laver à l’eau et au savon les tissus qui ont servi à couvrir les objets pendant la pulvérisation.	Fini minuw kera ka minnen girimaw datugu, o bee be ko ka je ni safine ye.	Wash with soap and water fabrics that were used to cover objects during spraying.
Laisser les portes et les fenêtres fermées pendant au moins 2 heures après la PID.	Fiyeli bane ko fe, aw be daw ani finetiriw tugulen to, fo ka tase lere fila ma.	Keep doors and windows closed for at least 2 hours after the spray.
Eviter de peindre, de mettre de l’enduit et de laver les murs pour préserver l’efficacité de l’insecticide.	So fiyelen kofe, aw man ka ka sow konona mu walima ku pintiri walima ku ko, walasa fennenamafagalan fanga kana ban.	Avoid painting, plastering, or washing the walls to preserve the effectiveness of the insecticide.

Abbreviations: PID, pulvérisation intradomiciliaire (indoor residual spraying); USAID, U.S. Agency for International Development.

After recording the messages, the team uploaded the voice recordings into the VOTO Mobile platform, a web-based platform that allows users to create and send voice messages. A benefit of having done the enumeration of beneficiary phone numbers was that the numbers were divided by geography and preferred language, as well as their preferred time to receive messages. Thus, the team was able to select the appropriate prerecorded message for each village and send them to the appropriate phone numbers. Upon answering the phone, the beneficiary would immediately hear the message about the IRS campaign. The team set the VOTO Mobile preferences to retry phone numbers up to 5 times. If, on the fifth attempt, a subscriber did not pick up their phone, the platform left the recording as a message. The team did not have the ability to check whether the voicemail had been listened to.

### Data Collection During IRS Implementation

Typically, spray operators collect specific information on the households receiving IRS. This information includes number of structures, total population residing in the structures, and reasons for not receiving IRS. For this pilot, in addition to the traditionally collected information, data on structure preparedness were collected in both intervention and comparison areas via a question that was added to the data collection form used by spray operators: Was the structure prepared to receive spraying?

The answer to this question indicates whether the beneficiaries had properly prepared before IRS by removing all household and food items from the structure and acts as means for estimating the effectiveness of mobilization in preparing structures. Structures that are not prepared in advance lead to delays ranging from 1 hour to an entire day, which can be very costly on a service delivery project such as IRS. Spray operators were trained to answer this question on their forms on their first approach to a structure.

### Beneficiary Perception Survey

After the IRS campaign was completed, the AIRS Mali M&E team wanted to understand the beneficiaries’ perception of text and voice messages as a means of IRS mobilization. In the pilot villages, the mHealth team went door to door, surveying 673 beneficiaries, as to how many messages residents received, if they understood the messages, and if the messages were helpful.

### Data Analysis

Once the data from these different activities were collected, we conducted several kinds of analysis to evaluate how the pilot performed compared with the door-to-door approach. We used Microsoft Excel to look at beneficiary literacy levels, quantity of voice and text messages sent, and perception of mobile messages across pilot villages. Similarly, Excel was used to analyze spray coverage and structure preparedness, the main indicator for evaluating effectiveness, in addition to creating the cost tracking spreadsheets used to perform cost analysis. Finally, to test the statistical significance in terms of preparedness differences among pilot and control villages, STATA 12 was used to perform Poisson regressions.

## RESULTS

### Literacy and Mobile-Messaging Preferences of Pilot Participants

**FIGURE 3:**
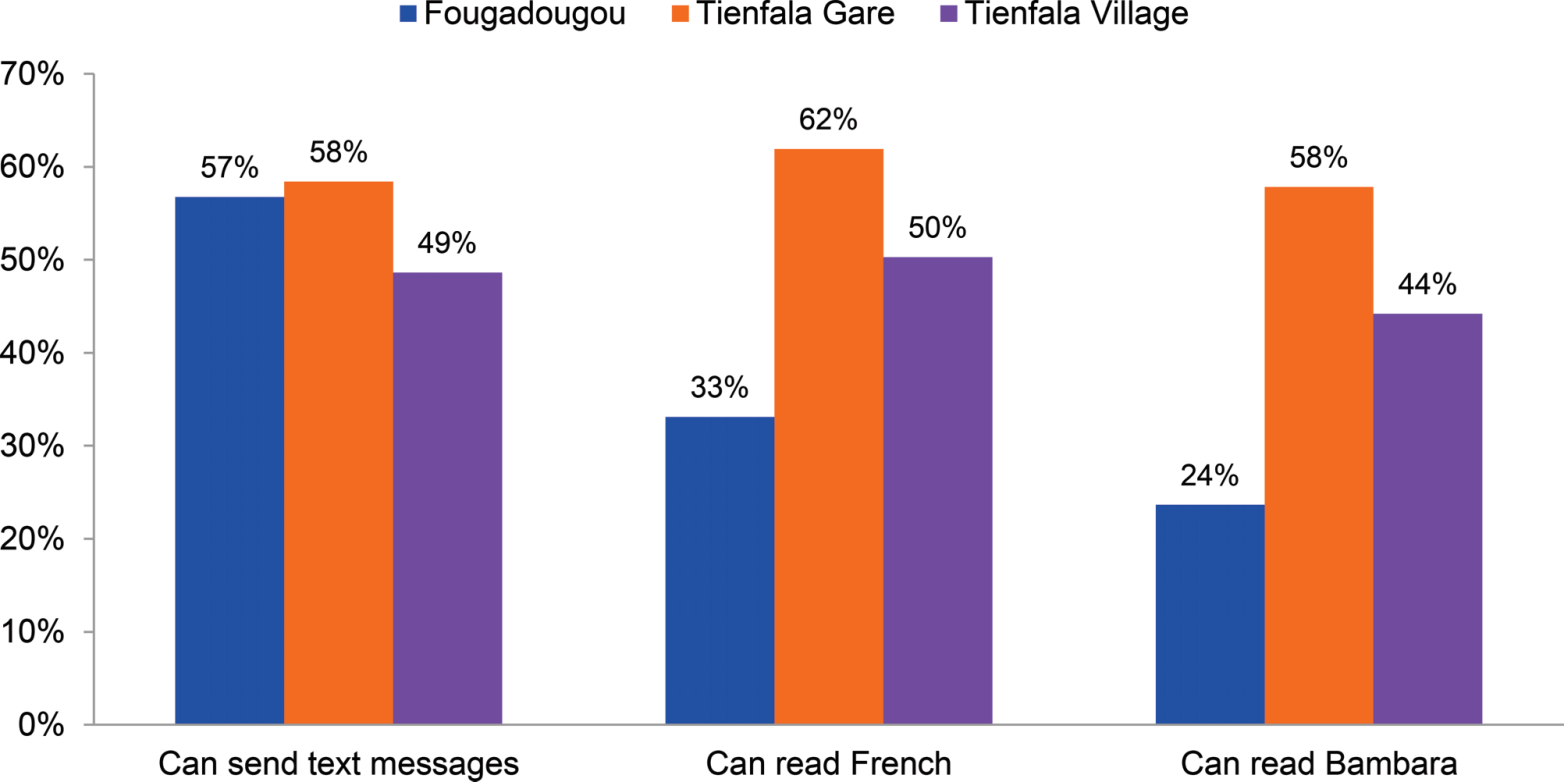
Literacy and Text-Messaging Capabilities of Beneficiaries, Koulikoro District, Mali, May–September 2014 (N=673)

Literacy in French and Bambara is relatively low in Fougadougou (33% and 24%, respectively), and only moderately better in Tienfala Gare (62% and 58%, respectively) (Figure 3). Levels of literacy in Tienfala Village fell in between the other 2 pilot villages, with 50% of respondents able to read in French and 44% able to read in Bambara.

The enumerators also questioned the beneficiaries about what time of day they preferred to receive text messages. The majority (65%) of beneficiaries reported preferring to receive messages in the afternoon or at night while 13% preferred to receive messages in the morning. About one-fifth (22%) of beneficiaries did not have a preference.

### Number of Messages Sent

The project sent 6,234 text messages to 673 beneficiaries in the 3 villages, or, on average, about 10 messages per beneficiary phone number. In the 2 villages that received voice messages in addition to the text messages, the project sent 4,474 voice messages to 477 beneficiaries, amounting to, on average, about 9 voice messages per beneficiary number (Table 2).

**TABLE 2:** Number of Messages Sent During the Pilot Indoor Residual Spraying Campaign, Koulikoro District, Mali, May–September 2014

**Village**	**Types of Messages Sent**	**No. of Beneficiary Phone Numbers**	**No. of Text Messages Sent**	**No. of Text Messages Sent per Beneficiary Phone Number**	**No. of Voice Messages Sent**	**No. of Voice Messages Sent per Beneficiary Phone Number**
Tienfala Village	Text only	181	1,760	10	0	0
Tienfala Gare	Voice + Text	344	3,290	10	3,290	10
Fougadougou	Voice + Text	148	1,184	8	1,184	8
**Total**		**673**	**6,234**	**9.3 (mean)**	**4,474**	**9.1 (mean)**

### Structure Preparedness and Spray Coverage

Structure preparation was significantly lower in households mobilized via the mobile-messaging approach compared with households mobilized using the door-to-door approach (49% vs. 75%, respectively; *P* = .03) (Table 3). In the end, both pilot and comparison villages had high spray coverage (defined as the percentage of structures identified during enumeration that were sprayed). However, spray coverage in pilot villages was lower compared with coverage in the villages mobilized through traditional door-to-door mobilization (85% vs. 96%, respectively; *P* = .02) (Table 3). It is also worth noting that mop-up teams had to be sent back to the pilot villages receiving mobile messaging to increase the number of structures accepting to be sprayed from the initial 80% coverage to the final 86%.

Structure preparation and spray coverage were significantly lower in households mobilized via mobile messaging vs. door-to-door mobilizers.

**TABLE 3:** Indoor Residual Spray Coverage and Household Preparedness in Pilot and Comparison Villages, Koulikoro District, Mali, May–September 2014

Village	No. of Structures Targeted	No. of Structures Sprayed	Spray Coverage	No. of Structures Prepared	Structure Preparation Coverage
**Mobile-Messaging Mobilization**					
Fougadougou	163	136	83%	66	40%
Tienfala Gare	262	212	81%	144	55%
Tienfala Village (text only)	151	140	93%	71	47%
**Subtotal**	**576**	**488**	**85%**	**281**	**49%**
**Door-to-Door Mobilization**					
Fassa	255	249	98%	199	78%
Wolongotoba Socoro	372	342	92%	230	62%
Wolongotoba Socoura	250	247	99%	232	93%
**Subtotal**	**877**	**838**	**96%**	**661**	**75%**

The most common reason structures were not sprayed in the pilot mobile-messaging areas was due to refusal. Other reasons cited by beneficiaries for not having their structures sprayed included sickness, closed/locked structure, and funeral in the household.

### Beneficiary Perceptions

In general, surveyed beneficiaries in the mobile-messaging areas were very curious about the mobile messages and wanted further information about the spray campaign. Interest was driven due to 2 main aspects of the mobile-messaging campaign. First, the number used to send the messages was based in the United States, and as such, beneficiaries wanted to verify the source of the number and were curious when seeing a text message from a foreign country code. Second, for people who had received voice messages, beneficiaries recognized the voices on the prerecorded message and wanted to ask those people further questions. In fact, many residents tried to speak to the voice recording or call back the number. Moreover, village residents who had received only text messages found out that other villages were receiving voice messages and wanted to know why they had not received the voice messages. They requested they be sent voice messages in addition to text messages.

Other qualitative information gained through interviews with beneficiaries related to how mobile messaging impacted men and women differently. For example, women felt they were no longer a part of the mobilization process. With door-to-door mobilization, women usually interacted with mobilizers. With mobile messaging, however, the men of the household more often received the information directly because they typically own the phones. Conversely, men enjoyed receiving the mobile messages because it provided them with the information directly, even if they were in the field working.

Women in the mobile-messaging areas felt excluded from the mobilization process since men typically owned mobile phones.

### Cost Analysis

An important driver for this pilot was to find ways to increase IRS mobilization efficiency while reducing implementation costs. This section analyzes the costs of the mobile-messaging and the door-to-door approach and then compares the costs to performance results in the different villages. Operational set-up costs, variable costs, and fixed costs for each approach were considered. Finally, per-structure costs are presented to evaluate the cost of these approaches if they were rolled out across all intervention areas in the AIRS Mali program as well as to allow a fair comparison of the 2 approaches, even though there were more structures in the comparison villages than the intervention villages.

#### Mobile-Messaging Mobilization Costs

To determine the cost per structure for the 2 different arms of the mobile-messaging approach—text only and voice plus text—we first considered the general set-up costs and the specific messaging costs separately. These were then combined to create the per-structure costs of each approach.

**Set-up costs.** The set-up costs associated with the mobile-messaging mobilization approach included the costs related to training enumerators, the enumeration of cell phone numbers and a data entry clerk, per diem costs for DTC supervision of data collection, Internet and phone card costs (fixed cost), and the costs of the mop-up, or the process of revisiting unsprayed structures (Table 4). The cost per structure for general set-up expenses was US$1.13, based on the 576 structures sprayed in all 3 villages. There were significant costs associated with mop-up for the mobile-messaging approach. It should be noted that in the 3 comparison villages the teams were able to spray all targeted structures during their first visit and so mop-up was not necessary.

The mobile-messaging approach included significant mop-up campaign costs while mop-up was not necessary in the door-to-door mobilization areas.

**TABLE 4:** Operational Costs for Mobile-Messaging Mobilization for the Pilot Indoor Residual Spraying Campaign, Koulikoro District, Mali, May–September 2014

Description	No. of People	No. of Days	Unit Cost (XOF)	Total Cost (XOF)	Total Cost (USD)
**Training**					
Enumeration training	5	1	10,000.00	50,000.00	100.00
Food during training	12	1	3,500.00	42,000.00	84.00
**Enumeration**					
Data collection in the field	4	3	1,500.00	18,000.00	36.00
DTC supervision (per diem)	1	5	10,000.00	50,000.00	100.00
Data entry clerk	1	10	8,000.00	80,000.00	160.00
**Operational Costs for TextIt/VOTO Mobile**^a^					
Software services	1	-	-	612.24	1.22
**Mop-Up (revisiting unsprayed structures in all 3 villages)**					
Spray operators	5	3	3,000.00	45,000.00	90.00
Team leader	1	3	3,500.00	10,500.00	21.00
Supervisor	1	3	5,000.00	15,000.00	30.00
DTC health official	1	3	5,000.00	15,000.00	30.00
**GRAND TOTAL**				**326,112.24**	**652.22**

Abbreviations: DTC, Health Center Technical Director; USD, U.S. Dollar; XOF, CFA Franc.

a These costs were estimated taking the overall costs associated with running the TextIt/VOTO Mobile applications and dividing it across all 735 villages in the project intervention areas that would be included in a full project roll out. This number was then multiplied by 3 to estimate the costs for the 3 treatment villages.

**Costs of text and voice messaging.** Costs differed among the pilot villages according to the type of message a village received because text messages were less expensive than voice messages. As such, a per-structure cost was calculated for Tienfala Village to represent the model of text messages only. This was calculated by multiplying the number of text messages sent by the cost of a text message (an average of the costs to send a text message on the same carrier and the costs to send between different carriers; US$0.05 per text message) and dividing by the number of structures mobilized within that village. Thus the per-structure cost of sending text messages was US$0.58.

Next, a per-structure cost was calculated for the villages that received both voice and text messages by multiplying the number of text messages sent by the cost of a text message, adding that to the multiplication of the number of voice messages sent by the cost of a voice message, including the total cost of recording the voice messages for both villages, and dividing the sum of those components by the total number of structures mobilized in both Tienfala Gare and Fougadougou. This resulted in a per-structure cost for sending both voice and text messages of US$3.89.

**Overall costs.** Finally, we compared the difference in per-structure costs for the village that received only text messages as compared with villages that received voice and text messages, as shown in the last column of Table 5. For Tienfala Village that received only text messages, the overall costs totaled US$1.71 per structure, while for the villages receiving both kinds of messages, the per-structure cost was US$4.73. It is important to note that there were no costs to the beneficiaries associated with receiving the mobile messages.

**TABLE 5:** Cost of Sending Text and Voice Messages per Structure for the Pilot Indoor Residual Spraying Campaign, Koulikoro District, Mali, May–September 2014

Village	No. of Structures Identified^a^	No. of Text Messages Sent	No. of Voice Messages Sent	Total Cost of Text and Voice Messages (USD)>	Voice Recording Costs (USD)	Cost of Messages per Structure (USD)	Operational Cost per Structure (USD)	Total Cost^b^ per Structure (USD)
Tienfala Village (text only)	151	1,760	0	88.00	0.00	0.58	1.13	1.71
Tienfala Gare and Fougadougou (text + voice)	425	4,474	4,474	1,207.98	320.00	3.60	1.13	4.73

a By spray operators during the first week.

b Comprises costs of sending text and voice messages and recording voice messages as well as operational costs.

#### Door-to-Door Mobilization Costs

During this pilot, the traditional door-to-door mobilization approach using interpersonal communication between mobilizers and households included additional aspects comprising radio spots and town hall announcements. By doing so, the team was able to reduce the number of mobilizers to 1 per village (previous campaigns used up to 3 mobilizers per village) and delayed the beginning of mobilization to just 10 days before the start of the IRS campaign. This approach has been adapted in many other PMI AIRS countries due to its continued effectiveness at reducing costs. The door-to-door mobilization approach included costs for training, per diems for various staff attending trainings, supervisory staff, and for fieldwork, radio spots, and lodging and transportation costs for associated personnel (Table 6).

**TABLE 6:** Costs of Door-to-Door Mobilization for the Pilot Indoor Residual Spraying Campaign, Koulikoro District, Mali, May–September 2014

Description	No. of People or Units	No. of Villages	No. of Days	Unit Cost (XOF)	Total Cost (XOF)	Total Cost (USD)
**FIXED COSTS**						
Per diem for DTC for IEC TOT	1	3	3	1,071.43^a^	9,643	19.29
DTC accommodation	1	3	3	1,428.57	12,857	25.71
District coordinator	1	3	3	159.36^b^	1,434	2.87
Per diem for NMCP/DNACPN staff	3	3	3	159.36^c^	4,303	8.61
Abt staff for IEC TOT	1	3	3	646.03^d^	5,814	11.63
Meals	1	3	3	1,145.00	10,302	20.60
Logistics IEC TOT - car rental	1	3	3	874.83	7,873	15.75
Logistics IEC TOT - fuel	1	3	3	336.47	3,028	6.06
Venue hire	1	3	3	201.88	1,817	3.63
Communication fees (subscription)	1	3		423.96	1,272	2.54
Office supplies	-	-	-	-	25,000	50.00
Radio spots	600^e^			13.46	8,075	16.15
**Subtotal**				**6,460.36**	**91,418**	**182.84**
**VARIABLE COSTS**						
Mobilizer training costs	1	3	3	15,000.00	135,000	270.00
Mobilizer daily salary	1	3	10	1,500.00	45,000	90.00
Per diem for DTC supervision	1	3	1	10,000.00	30,000	60.00
Town crier	1	3	15	1,000.00	45,000	90.00
**Subtotal**					**255,000**	**510.00**
**GRAND TOTAL**					**346,418**	**692.84**

Abbreviations: DNACPN, Direction Nationale de l'Assainissement et du Contrôle des Pollutions et des Nuisances (National Directorate for Sanitation and Pollution Control); DTC, Health Center Technical Director; IEC, information, education, and communication; NMCP, National Malaria Control Program; TOT, training of trainers; USD, U.S. Dollar; XOF, CFA Franc.

a For DTC-related expenses, their unit costs were calculated by dividing the normal cost for 1 DTC supervisor by 14 villages. DTC personnel supervise 14 villages on average.

b The expenses for district coordinators were divided across all 251 villages in Koulikoro District.

c The NMCP/DNACPN staff covered the entirety of Koulikoro District; thus, their cost was divided by Koulikoro’s 251 villages.

d Total cost for the Abt staff, meals, car rental, fuel, venue hire, and radio spots were divided across all 735 in the project intervention areas in order to arrive at the unit costs listed.

e This number is based on 30 different radio spots that were broadcast over 20 different radio stations.

The overall cost of this door-to-door approach was US$692.84, which covered 877 structures in 3 villages, resulting in a per-structure cost of US$0.79.

#### Mobile-Messaging vs. Door-to-Door Mobilization Costs

The cost analysis revealed that the per-structure cost of the door-to-door mobilization approach was less expensive than either of the mobile-messaging approaches. Mobile messaging using only text messages was 3.4 times as expensive per structure prepared as the door-to-door approach, and mobile messaging using both voice and text messaging was more than 7 times more expensive per structure prepared than the door-to-door approach.

Mobile messaging was 3–7 times more expensive than traditional door-to-door mobilization.

It is also important to look at the relative performance of each approach to determine which method was more effective. Table 7 shows the calculations of U.S. dollars spent per structure prepared for each village: the number of structures identified by spray operators in the village multiplied by the cost of mobilization per structure for the method used in that village. This cost was then divided by the actual number of houses that were prepared when the spray operators arrived.

**TABLE 7:** Cost per Structure Prepared for the Pilot Indoor Residual Spraying Campaign, Koulikoro District, Mali, May–September 2014

Village	No. of Structures Targeted	Mobilization Costs (USD)	No. of Structures Prepared	% Structures Prepared	Cost per Structure Prepared (USD)
**Door-to-Door Mobilization Villages**					
Fassa	255	201.45	199	78.04%	1.01
Wolongotoba Socoro	372	293.88	230	61.83%	1.28
Wolongotoba Socoura	250	197.50	232	92.80%	0.85
**Mobile-Messaging Mobilization Villages**					
Fougadougou	163	771.00	66	40.49%	11.68
Tienfala Gare	262	1,239.26	144	54.96%	8.61
Tienfala Village (text only)	151	258.21	71	47.02%	3.64

Mobile-messaging mobilization was both more costly and less effective at preparing structures to be sprayed. In areas that used mobile-messaging mobilization, AIRS Mali spent US$8.17 (a weighted average of the costs across all 3 villages) per structure prepared, while in the modified door-to-door mobilized areas, the project spent US$1.08 (a weighted average of the costs across all 3 villages) per structure prepared.

## DISCUSSION

The villages that received mobile-messaging mobilization had higher refusal rates for spraying their houses with insecticide and were not as prepared as the comparison group of villages that received the modified door-to-door mobilization with radio spots and town hall announcements. Despite using best practices for the mobile-messaging mobilization activities, such as thorough enumeration to gather phone numbers and testing the text messages before the start of the spray campaign, the pilot intervention was not as effective as door-to-door mobilization. One reason could be that text and voice messages are a new technology for most beneficiaries, and so while many residents in the pilot villages owned phones, they did not use them regularly. Furthermore, this was the first time many of the subscribers had received a public health mobilization message through their mobile device. Future mHealth communication campaigns may experience better results once beneficiaries are more familiar with this mobile-messaging mobilization concept. However, mobile messaging might not be able to completely replace interpersonal communication between mobilizers and households. When beneficiaries were hesitant to have their structures sprayed (e.g., not wanting to miss a day of farming or not wanting to take out all their furniture), it was much easier to ignore the mobile message. On the other hand, when a mobilizer goes door-to-door on the day of spray, it is harder for beneficiaries to ignore the mobilizer because there is an opportunity to have their questions answered and build trust, creating more tangible motivation to accept having their structures sprayed. In person, the mobilizer can work directly with the beneficiary or village leader to gain acceptance. Finally, it should also be considered that the literacy rate is quite low in Mali; in the districts where IRS is implemented in the country, the literacy rate is 33.6%.^6^

It is much easier for people to ignore mobile phone campaign messages than mobilizers at their door.

### Lessons Learned

1. Mobile-messaging mobilization in Mali had limited effectiveness because much of the population is illiterate and thus could not read the IRS mobilization text messages. Some illiterate residents, however, would ask other individuals to read the messages to them allowing some illiterate beneficiaries to still get the message about the timing of IRS. This illiteracy was also not overcome through the addition of voice messages in 2 of the 3 pilot villages. Implementation of the mobile-messaging mobilization approach in countries with higher literacy rates could potentially increase comprehension and utility of these messages. A potential list of countries with higher literacy rates for the population between 15 and 65 years old and where the AIRS PMI Project works includes: Ghana (76.6%), Madagascar (64.7%), Rwanda (70.5%), Zambia (63.4%), and Zimbabwe (86.5%).^7^
2. It is much easier for beneficiaries to ignore a text message—and even a voice message—than a face-to-face interaction in regards to preparing their household for spraying. With a text or voice message, beneficiaries may have the good intentions to comply, but could easily forget once they put their phone down. However, if there is someone there in person asking household members to comply, it will be harder to forget or ignore this request.
3. Although the enumeration was very important for this pilot because it let the AIRS Mali team test the technology before implementation, it has proven to be a very costly exercise and would likely have to be done each year, as cell phone numbers change frequently.
4. After analyzing aspects of the door-to-door approach, it became clear that the additions of radio announcements and community town hall meetings helped to ensure that mobilization messages reached a large portion of the community. Moreover, the community meetings helped communicate important messages, such as the change in insecticide and the description of the pilot project, and they reinforced the benefits of IRS. If radio announcements and town hall meetings had been added to the mobile-messaging approach, the team could potentially have seen better structure preparedness because it would have added an important human aspect to an approach that otherwise lacks traditional social interaction.
5. The use of the TextIt platform was not the most economical choice as it is a system based in the United States. Future interventions attempting to implement similar mobile-messaging approaches should investigate possible options of partnering with locally based telecom providers, to reduce the overall costs associated with sending mass messages. Similarly, use of an open-source mass text messaging service could reduce the associated costs of running such software.
6. This pilot demonstrated that different methods of mobilization can affect men and women differently. With door-to-door mobilization, women usually interact with mobilizers since they are typically at home at the time when mobilizers visit. With mobile messaging, however, the men of the household more often received the information directly because they typically own the phones. This is a significant difference in approach because women are usually at home during the day to allow the IRS team to enter the household. By relying only on mobile messaging, the team no longer engaged women and this could be seen as an unanticipated effect of choosing an mHealth approach.

Radio announcements and town hall meetings were an important addition to the traditional door-to-door IRS mobilization approach.

### Implications for Future mHealth Projects

While the results of this pilot do not suggest relying solely on text or voice messages for IRS mobilization in the same implementation design as was done in Mali, it does provide a stepping stone toward potentially more effective implementation of mHealth approaches. The use of mHealth has been found to be most effective in producing health outcomes when incorporated as part of a multifaceted behavior change strategy, as opposed to a stand-alone intervention. For instance, mobile-messaging mobilization could be used in combination with a mobilizer traveling with an IRS team on the day of spraying. This pilot did not choose to test a hybrid blend of mobile messaging and standard mobilization approaches since the team’s goal was to evaluate a unique mobile-message approach, but a hybrid approach could be tested in future campaigns. The 2 methods of communication (mobile and interpersonal) complement each other, but the population in these villages is not yet comfortable enough with mobile technology to rely solely on mobile-based messaging.

There are advantages to using mobile-based mobilization, such as the ability to alert a village remotely the morning of arrival of IRS teams. One drawback is the higher cost of the mobile-messaging approach compared with the traditional approach, in part a result of the additional burden of a separate phone enumeration process that would theoretically have to be repeated each year. A possible way of reducing this cost moving forward is to incorporate phone number collection into the spray operators’ regular data collection processes. Thus, after the first year of IRS implementation, the team would have phone numbers for each structure that could then be used in upcoming IRS campaigns.

## CONCLUSION

This case study provides a useful example where an mHealth approach was *not* appropriate given contextual constraints and highlights the importance of performing a contextual analysis before choosing to move forward with using mHealth for public health interventions. As noted earlier, the low literacy and strong dependency on human interaction and social ties in Mali do not create an atmosphere conducive to such a technological innovation. By eliminating the human interaction aspect of mobilization through the mobile-messaging approach, the overall success of IRS mobilization suffered due to lower rates of acceptance and structure preparedness. If key components such as high literacy and familiarity with newer technology are available, combining mHealth with a broader public health intervention could produce fruitful results.

## Notes

**Peer Reviewed**
